# Identification of Pharmacologically Tractable Protein Complexes in Cancer Using the R-Based Network Clustering and Visualization Program MCODER

**DOI:** 10.1155/2017/1016305

**Published:** 2017-06-13

**Authors:** Sungjin Kwon, Hyosil Kim, Hyun Seok Kim

**Affiliations:** ^1^Graduate Programs for Nanomedical Science, Yonsei University, Seoul, Republic of Korea; ^2^Severance Biomedical Science Institute, Brain Korea 21 Plus Project for Medical Science, Yonsei University, College of Medicine, Seoul, Republic of Korea

## Abstract

Current multiomics assay platforms facilitate systematic identification of functional entities that are mappable in a biological network, and computational methods that are better able to detect densely connected clusters of signals within a biological network are considered increasingly important. One of the most famous algorithms for detecting network subclusters is Molecular Complex Detection (MCODE). MCODE, however, is limited in simultaneous analyses of multiple, large-scale data sets, since it runs on the Cytoscape platform, which requires extensive computational resources and has limited coding flexibility. In the present study, we implemented the MCODE algorithm in R programming language and developed a related package, which we called MCODER. We found the MCODER package to be particularly useful in analyzing multiple omics data sets simultaneously within the R framework. Thus, we applied MCODER to detect pharmacologically tractable protein-protein interactions selectively elevated in molecular subtypes of ovarian and colorectal tumors. In doing so, we found that a single molecular subtype representing epithelial-mesenchymal transition in both cancer types exhibited enhanced production of the collagen-integrin protein complex. These results suggest that tumors of this molecular subtype could be susceptible to pharmacological inhibition of integrin signaling.

## 1. Introduction

Biological functions often arise from multisubunit protein complexes, rather than a single, isolated protein [1, 2]. Many high throughput assay platforms in genomics, transcriptomics, and proteomics have become standard methods for investigating gene/protein interactions that give rise to biological functions [3]. However, because of biological and technical errors, these methods are hindered by a limited signal-to-noise ratio, rendering them vulnerable to high rates of false positives and false negatives; particularly when discovered hits represent a single gene, protein, and so forth. In this regard, codiscovery of hits for multiple subunits of a protein complex in an experimental condition helps mutually support the significance of such findings [4]. Detection of higher order clusters in a large network, however, is computationally challenging [5]. A number of algorithms have been developed over the past decade to tackle this problem, including the Markov Cluster Algorithm (MCL) [6], Molecular Complex Detection (MCODE) [7], DPClus [8], Affinity Propagation Clustering (APC) [9], Clustering based on Maximal Clique (CMC) [10], ClusterMaker [11], and Clustering with Overlapping Neighborhood Expansion (ClusterONE) [12]. Many of these algorithms have been implemented in various Cytoscape applications (CytoCluster, ClusterViz [13], and ClusterMaker [11]), as well as in java-based applications (C-DEVA [14]). Of these, as of February 2017, MCODE was the most downloaded Cytoscape application within the clustering category. MCODE discovers interconnected network clusters based on *k*-core score: the *k*-core of a particular graph (graph X) represents the maximal number of connected subgraphs of graph X, in which all nodes are connected by *k* (minimum number of degrees). Although Cytoscape is a java-based, open source, bioinformatics software platform with a user-friendly graphic-user interface [15], it requires extensive computational resources due to the memory restraints of java virtual machines (Cytoscape version 3.2.1: 2 GB+ recommended). Thus, its capacity to process input networks and graphical outputs is limited. For a computationally intensive task, R may be a better-suited platform. R is the most popular open source, statistical programming language, and data analysis platform used in analysis of broad, high throughput, and multiomics data. While the platform is suitable for iterative analysis of large-scale data sets in batch mode, R-based network clustering software is rare. Herein, we describe our implementation of the MCODE algorithm in R programming language and a related package, hereinafter referred to as MCODER. The MCODER package can be easily integrated into custom R projects and provides powerful and enhanced graphical output options, compared to its Cytoscape counterpart.

The Cancer Genome Atlas projects have classified tumors into subtypes that share distinct molecular and genetic features. To do so, researchers have leveraged multiomics data sets, including global and phosphoproteomic quantification, as well as DNA- and RNA-level measurements. Nevertheless, drawing associations between these subtypes and clinically important features, such as prognosis and therapeutic options, remains important challenges. In this study, we intended to focus on these challenges in high-grade serous ovarian carcinoma (HGS-OvCa) and colorectal cancer (CRC). Currently, standard treatment for ovarian cancer involves primary cytoreductive surgery, followed by platinum-based chemotherapy. Only two targeted therapies are clinically available for ovarian cancer, including poly (ADP-ribose) polymerase inhibitors and angiogenesis inhibitors in recurrent ovarian cancer [16], although they have been shown to offer little survival benefit. The four molecular subtypes of HGS-OvCa are differentiated, immunoreactive, proliferative, and mesenchymal, according to gene content analysis within each subtype, following transcriptome-based subtype classification [17, 18]. Of these, the mesenchymal subtype displays the worst prognosis [19, 20]. Meanwhile, CRC has four consensus molecular subtypes (CMSs): CMS1, CMS2, CMS3, and CMS4. The CMS subtypes of CRC are associated with various clinical features, such as sex, tumor site, stage at diagnosis, histopathological grade, and prognosis, as well as molecular features of microsatellite status, CpG island methylator phenotype (CIMP), somatic copy number alteration (SCNA), and enrichment of particular driver mutations. The CMS1 subtype exhibits high MSI, high CIMP, strong immune activation, and frequent* BRAF* mutation and involves an intermediate prognosis, showing worse survival after relapse. The CMS2 subtype displays a high degree of chromosomal instability (high SCNA), frequent* APC* mutation, and good prognosis. Tumors of the CMS3 subtype display mixed MSI, high SCNA, frequent* KRAS* mutation, metabolic deregulation, and good prognosis. Finally, the CMS4 subtype is characterized by distinct epithelial-mesenchymal transition (EMT) signature, high SCNA, and the poorest prognosis. Notably, in both cancers, mesenchymal subtype confers the worst prognosis. To gain insights into molecular subtype-selective opportunities for targeted therapies in ovarian and colorectal cancer, HGS-OvCa [21] and CRC [22] data sets were analyzed using MCODER. Both data sets contained mass-spec-based quantitative proteomic assay results for the well-defined molecular subtypes of these cancers. In particular, we aimed to identify pharmacologically tractable protein complexes selectively elevated within the distinct molecular subtypes of both cancers.

## 2. Implementation

MCODER identifies the maximal subset of vertices interconnected by the minimal number of degrees (*k*) from an input network of nodes (genes or proteins) and edges (pairwise interactions). Although the MCODER package does not account for the direction of the edges when calculating *k*-core scores and when detecting subnetworks, it can indicate directions using arrows and display multiple edges between a pair of nodes, which is not supported by the original MCODE. Moreover, various graphical parameters provided by “igraph” (http://igraph.org/redirect.html) can be manipulated in MCODER, facilitating customization of the shape, size, and color of the network output. The MCODER R package requires preinstallation of two other packages, “sna” (Social Network Analysis) (https://cran.r-project.org/web/packages/sna/index.html) for calculating *k*-cores and “igraph” for plotting figures.

**Figure 1:**
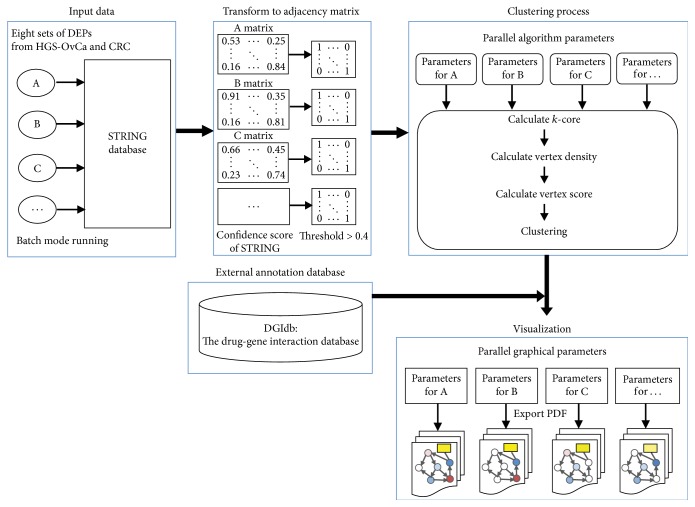
Workflow for detecting densely connected network clusters using MCODER. See Implementation for further details.

The overall workflow of the present study to identify pharmacologically tractable protein complexes is presented in Figure 1. Before running MCODER, we downloaded the STRING database (Homo sapiens, v10.0) from http://string-db.org: STRING is an archive of direct (physical) and indirect (functional) protein-protein interactions [23]. We filtered low confident interactions by applying an interaction-score cutoff (score < 0.4) to obtain 13,159 genes with 738,312 interactions. In parallel, we downloaded and preprocessed proteome data sets by selecting samples that have preassigned molecular subtypes and matched normal controls to obtain input data sets: HGS-OvCa (*n* = 3,329 proteins, 140 samples) and CRC (*n* = 3,718 proteins, 70 samples) [21, 22]. HGS-OvCa consisted of four molecular subtypes: differentiated (*n* = 35 samples), immunoreactive (*n* = 37 samples), proliferative (*n* = 34 samples), and mesenchymal (*n* = 34 samples). CRC consisted of four molecular subtypes: CMS1 (*n* = 14 samples), CMS2 (*n* = 28 samples), CMS3 (*n* = 9 samples), and CMS4 (*n* = 14 samples). To identify differentially expressed proteins (DEPs) selectively elevated in a particular molecular subtype, a one-sided *t*-test was conducted iteratively within a tumor (e.g., CMS1 versus CMS2, CMS3, CMS4). After preparing differentially expressed protein sets, we converted them into adjacency matrices for each set, with connection information between nodes according to the STRING database, followed by calculation of *k*-core values, vertex density, and vertex score. Self-loop and duplicated connections between nodes were not considered for the calculation. Clusters were detected with the following parameters: minimal *k*-core value = 2, haircut = TRUE, fluff = FALSE, self-loop = FALSE, node score cutoff = 0.2, depth = 20, and degree cutoff = 2. Subsequently, vertices in the clusters were annotated according to the DGI database [24], allowing for detection of druggable DEPs.

## 3. Results

First, we examined the performance of MCODER (Figure 1) in comparison to the MCODE Cytoscape application, testing input networks of different sizes (Table 1). All tests were performed using MacBook Pro (Mac OS X, Late 2013, 2.4-GHz Intel Core i5, 8 GB RAM). Input data sets were prepared by random sampling of the given number of interactions from the STRING database. We found that both software packages returned identical protein complexes as an output. Meanwhile, however, MCODER in the R environment offered enhanced performance in regard to speed and memory usage in all test settings (Table 1). The MCODER installation package is available online at https://sourceforge.net/projects/mcoder.

**Figure 2:**
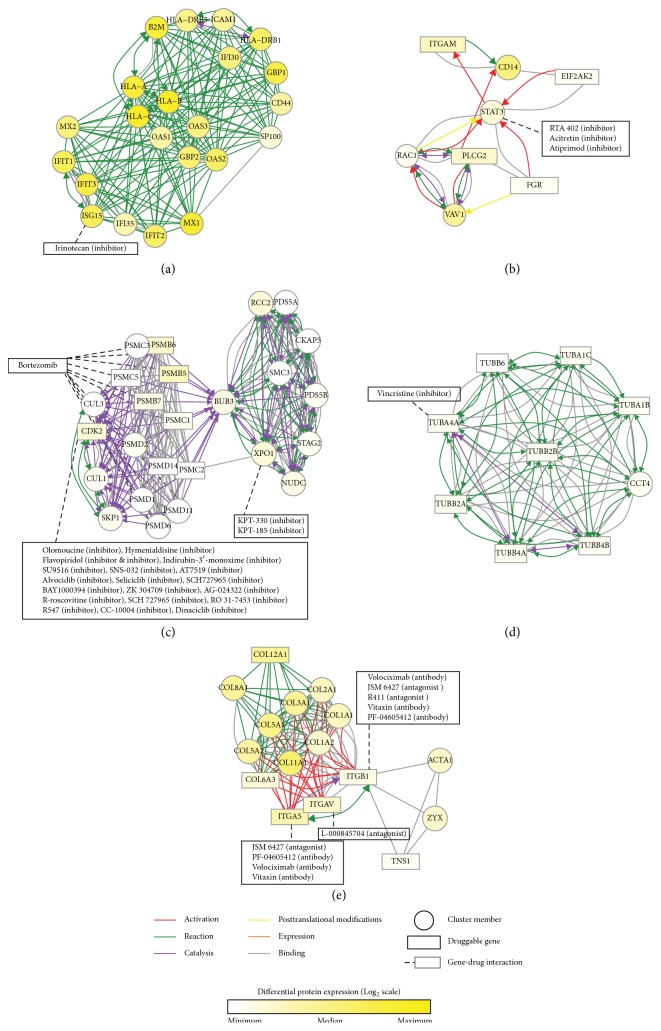
Pharmacologically targetable network clusters overexpressed in molecular subtypes of HGS-OvCa: (a, b) immunoreactive, (c, d) proliferative, and (e) mesenchymal subtype.

Next, for the individual molecular subtypes, we identified selectively elevated proteins under a *p* value threshold of 0.01: 300 proteins for differentiated, 284 proteins for immunoreactive, 547 proteins for proliferative, and 493 proteins for mesenchymal HGS-OvCa and 236 proteins for CMS1, 284 proteins for CMS2, 134 proteins for CMS3, and 137 proteins for CMS4 subtypes of CRC (see Supplementary Data 1 in the Supplementary Material available online at https://doi.org/10.1155/2017/1016305). For each of the DEP sets, MCODER identified highly interconnected subnetworks of protein-protein interactions. For HGS-OvCa, we detected pharmacologically targetable clusters in three of the four subtypes (Supplementary Data 2). In the immunoreactive subtype, two clusters showed connections with pharmacological agents. The first cluster contained interferon-stimulated gene 15* (ISG15)*, which is a biomarker for predicting sensitivity to irinotecan, an anticancer drug and topoisomerase I inhibitor (Figure 2(a)). Previous studies have demonstrated that* ISG15* encodes an ubiquitin-like protein conjugated to specific E3 ubiquitin ligases and seems to inhibit the signaling consequences of ubiquitin/26S proteasome pathways [25]. Currently, treatments with irinotecan, in combination with bevacizumab or cisplatin, are in clinical trials for recurrent ovarian cancer [26]. Our findings suggest that selecting patients with immunoreactive features might increase response rates to irinotecan in future trials. The second cluster comprised a chemokine signaling related protein complex, including STAT3, which can be inhibited by RTA402, acitretin, and atiprimod (Figure 2(b)). Previous studies have indicated that STAT3 inhibitors, in combination with cisplatin, enhance cisplatin sensitivity in cisplatin-resistant ovarian cancer [27, 28]. Thus, a combination of irinotecan and STAT3 inhibitors might be plausible in treating ovarian cancers of immunoreactive subtype. In the proliferative subtype, two clusters displayed connections with pharmacological compounds. The CDK2-proteasome-XPO1 cluster was enriched with pharmacological options, including the proteasome inhibitor bortezomib, which is available clinically, and CDK2 and XPO1 inhibitors, which are under active clinical trials for various tumor types (Figure 2(c)) [29, 30]: XPO1 inhibitors have been used to target platinum-resistant ovarian tumors [31] and have been described as potentially inhibiting abnormal NF-kB signaling [32]. The second DEP cluster was the tubulin complex, in which TUBA4A can be targeted by vincristine to blunt mitotic chromosomal separation (Figure 2(d)). Similar to paclitaxel, a microtubule stabilizer and an antiproliferative agent [33], vincristine may be a potential agent for the treatment of ovarian cancer, particularly that of proliferative subtype. In the mesenchymal subtype, focal adhesion, endocytosis, vascular smooth muscle contraction, the PI3K-AKT signaling pathway, and so forth were identified. Of these, the integrin-collagen complex is a pharmacologically tractable target; various integrin signaling inhibitors include ITGA5 inhibitors (JSM 6427, PF-04605412, Volociximab, and Vitaxin), ITGB1 inhibitors (Volociximab, JSM 6427, R411, Vitaxin, and PF-04605412), and an ITGAV inhibitor (L-000845704) (Figure 2(e)). Integrin signaling is involved in the migration, invasion, proliferation, and survival of cancer cells [34]. Recently published studies have demonstrated that integrins participate in maintaining cancer stem cell populations and contribute to cancer progression and drug resistance [35]. Although integrin inhibitors as monotherapy agents have failed to demonstrate benefits in metastatic ovarian tumors, possibly due to compensation by other integrins [36], simultaneous targeting of integrin-FAK and c-Myc signaling has been found to synergistically disrupt tumor cell proliferation and survival in HGS-OvCa [37], supporting the notion of combinatorial targeting of integrin as a valid approach for treating ovarian cancer, particularly that of mesenchymal subtype.

**Figure 3:**
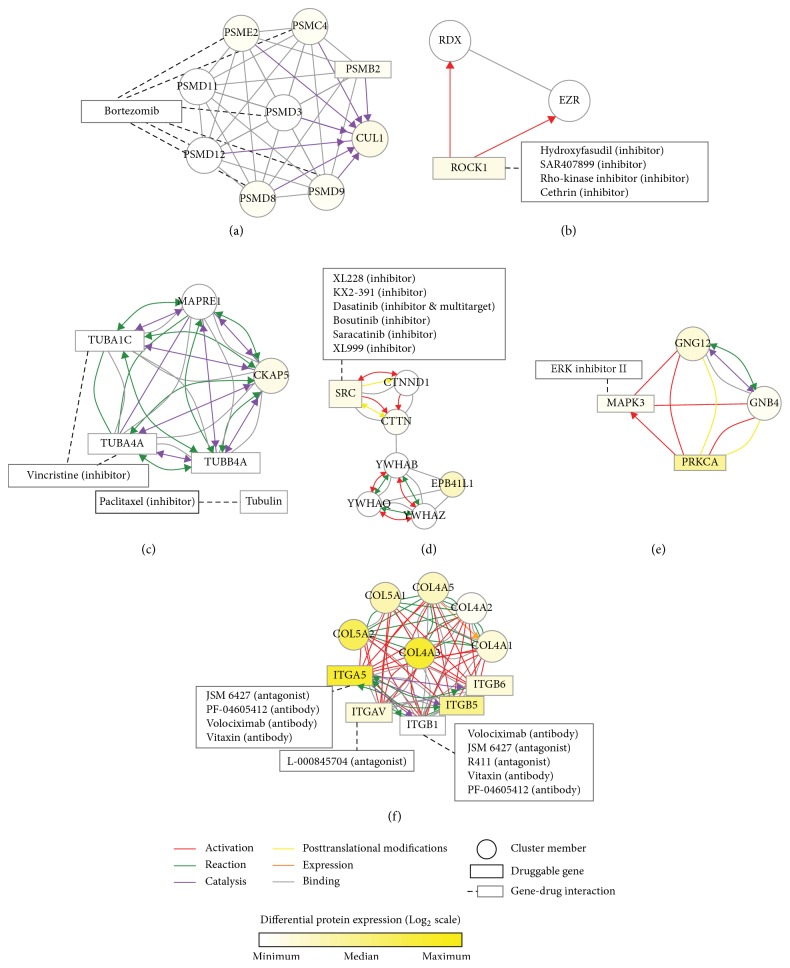
Pharmacologically targetable network clusters overexpressed in molecular subtypes of CRC: (a, b) CMS1, (c, d) CMS2, and (e, f) CMS4.

For CRC, MCODER identified pharmacologically targetable protein complexes in three of the four CMSs (Supplementary Data 2). In CMS1 subtype (MSI immune), proteasome complex (similar to the HGS-OvCa proliferative subtype) and ROCK1 signaling subnetworks were found to be overexpressed (Figures 3(a)-3(b)). Bortezomib treatment has been shown to induce G2-M arrest by activation of an ataxia-telangiectasia mutated protein-cell cycle checkpoint kinase 1 pathway in colon cancer cells [38]. Combination of platelet-derived growth factor and the ROCK inhibitor Y27632 has been found to decrease the invasive potential of SW620 colon cancer cells [39]. In the CMS2 subtype (canonical), tubulin complex was found to be elevated, similar to the HGS-OvCa proliferative subtype (Figure 3(c)). This observation suggests that vincristine could have therapeutic effects on CRCs of CMS2 subtype. Alternatively, or in combination with microtubule inhibitors, Src inhibitors may also be a plausible approach for CMS2 tumors (Figure 3(d)). The CMS4 subtype of CRCs exhibits EMT activation and confers the poorest prognosis. Other study groups have formerly referred to this subtype as colon cancer subtype 3 [40] or stem-like subtype [41]. In CMS4 tumors, we found the total MAPK3 (ERK1) protein complex to be elevated, which is targetable with ERK inhibitor II (Figure 3(e)). Surprisingly, in accordance with the HGS-OvCa mesenchymal subtype, CMS4 was also characterized by elevation of the extracellular matrix collagen-integrin complex (Figure 3(f)): collagen in the extracellular matrix has indeed been found to drive EMT in CRC [42]. Thus, the collagen-integrin protein complex may work as a molecular linchpin that, when removed, could diminish the malignant potential of EMT tumors. Accordingly, we suggest that therapeutic antibodies that interrupt the signaling of integrin proteins could potentially be utilized as therapeutic options, in combination with other chemo- or targeted therapies, for this refractory subtype of colon cancer.

Finally, we sought to determine whether our findings are reproducible with other network clustering algorithms, including ClusterONE [12] and MCL [6]. Although the sizes of the detected clusters varied, all of the subclusters detected by MCODER were identified by these algorithms as well, indicating that our findings are robust across different clustering algorithms.

## 4. Discussion

In this study, we implemented the network clustering algorithm MCODE into the R software environment (which we called MCODER) and demonstrated that the MCODER package saves computational resources and time, making it particularly suited for analyzing multiple omics data sets. Using MCODER, we identified potential candidates for anticancer therapy in molecular subtypes of ovarian and colorectal cancer by detecting protein complexes that were selectively overexpressed therein and that could be targeted with known pharmacological agents. For HGS-OvCa, we found that irinotecan and STAT3 inhibitors may be candidates for the immunoreactive subtype, along with bortezomib, CDK2, XPO1 inhibitors, and vincristine for the proliferative subtype and integrin signaling inhibitors for the mesenchymal subtype. For CRC, we found bortezomib and ROCK inhibitors to be potential candidates for the CMS1 subtype, along with vincristine and Src inhibitors for the CMS2 subtype and ERK inhibitor II and integrin signaling inhibitors for the CMS4 subtype. Importantly, our analyses revealed that the collagen-integrin protein complex, which is pharmacologically tractable, is commonly overexpressed in EMT subtypes of both ovarian and colorectal cancers. Further studies are needed to determine whether pharmacological inhibition of collagen-integrin signaling blunts tumor growth in an in vivo model of EMT cancer.

## Supplementary Material

Supplementary Data 1: List of differentially expressed proteins in each of the molecular subtypes of HGS-OvCa and CRC.
Supplementary Data 2: Identified submodules for each of the molecular subtypes of HGS-OvCa and CRC.

## Figures and Tables

**Table 1:** Comparison of computational time and memory usage between MCODER and the MCODE Cytoscape application.

Network size	Performance	
	MCODER	Cytoscape MCODE
5K edges, 2,902 vertexes	6 s.	1 m. 14 s.
100K edges, 3,786 vertexes	11 s.	3 m. 44 s.
200K edges, 4,625 vertexes	19 s.	18 m. 47 s.
Memory usage	0.45 GB	5 GB
